# Revisiting Special Relativity: A Natural Algebraic Alternative to Minkowski Spacetime

**DOI:** 10.1371/journal.pone.0051756

**Published:** 2012-12-31

**Authors:** James M. Chappell, Azhar Iqbal, Nicolangelo Iannella, Derek Abbott

**Affiliations:** School of Electrical and Electronic Engineering, University of Adelaide, Adelaide, South Australia, Australia; Universita’ del Piemonte Orientale, Italy

## Abstract

Minkowski famously introduced the concept of a space-time continuum in 1908, merging the three dimensions of space with an imaginary time dimension 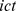, with the unit imaginary producing the correct spacetime distance 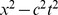, and the results of Einstein’s then recently developed theory of special relativity, thus providing an explanation for Einstein’s theory in terms of the structure of space and time. As an alternative to a planar Minkowski space-time of two space dimensions and one time dimension, we replace the unit imaginary 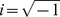, with the Clifford bivector 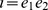 for the plane that also squares to minus one, but which can be included without the addition of an extra dimension, as it is an integral part of the real Cartesian plane with the orthonormal basis 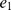 and 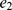. We find that with this model of planar spacetime, using a two-dimensional Clifford multivector, the spacetime metric and the Lorentz transformations follow immediately as properties of the algebra. This also leads to momentum and energy being represented as components of a multivector and we give a new efficient derivation of Compton’s scattering formula, and a simple formulation of Dirac’s and Maxwell’s equations. Based on the mathematical structure of the multivector, we produce a semi-classical model of massive particles, which can then be viewed as the origin of the Minkowski spacetime structure and thus a deeper explanation for relativistic effects. We also find a new perspective on the nature of time, which is now given a precise mathematical definition as the bivector of the plane.

## Introduction

It has been well established experimentally that the Lorentz transformations, provide a correct translation of space and time measurements from one inertial frame of reference to another. They were developed by Lorentz [1] with further refinements by Poincaré [2], [3], to explain the null result of the Michelson-Morley experiment, proposing a length contraction of a laboratory frame of reference moving with respect to a hypothetical aether [4]–[8]. Einstein, however, rederived the Lorentz transformations on the basis of two new fundamental postulates [9], of the invariance of the laws of physics and the invariance of the speed of light, between inertial observers, thus eliminating the need for an aether. Minkowski in 1908, however, also derived the Lorentz transformations from a different perspective, postulating a spacetime continuum, from which the results of special relativity also naturally followed [10], but which additionally provided a general structure for spacetime within which all the laws of physics should be described [11], [12]. Specifically, he introduced a four-dimensional Euclidean space with the expected Pythagorean distance measure 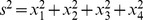, defining 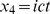, where 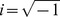 is the unit imaginary, which thus allowed one to view spacetime as a conventional Euclidean space with no difference in treatment between the 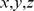 and 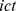 coordinates [13], [14], but still recovering the invariant distance measure 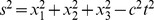. This idea was received favorably by Einstein, and by the wider scientific community at the time [15], but more recently, with the desire to remain consistent with the real metric of general relativity, the unit imaginary has been replaced with a four-dimensional metric signature 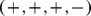 [16], [17].

In this paper however we propose an alternate spacetime framework to Minkowski, using the multivector of a two-dimensional Clifford algebra, replacing the unit imaginary representing an imaginary time coordinate, with the Clifford bivector 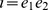 of the plane, defined by the orthonormal elements 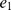 and 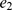, which also has the property of squaring to minus one. The bivector however has several advantages over the unit imaginary in that (i) it is a composite algebraic component of the plane and so an extra Euclidean-type dimension is not required and (ii) the bivector is an algebraic element embedded in a strictly real space, and hence consistent with the real space of general relativity. Clifford’s geometric algebra of two-dimensions can be adopted as a suitable algebraic framework to describe special relativity, because the Lorentz transforms act separately on the parallel and perpendicular components of vectors relative to a boost direction thereby defining a two-dimensional space.

Clifford algebra has been used previously to describe spacetime [18]–[21], however these approaches follow Minkowski in describing a four-dimensional spacetime framework with an associated mixed metric, such as the STA of Hestenes [18] which uses the four algebraic non-commuting basis elements 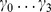, with 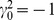 representing the time dimension and 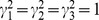 for space. In order to relate these definitions to our framework, we can make the identifications 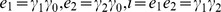. However the STA framework in two dimensions requires three unit vectors, as opposed to two in our approach, as well as the requirement for a mixed metric. A related approach by Baylis [22], called the Algebra of Physical Space (APS), in two dimensions involves just two space unit vectors that are added to a scalar variable representing time, that is 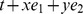. This is an effective approach, though we now need to define a special form for the dot product in order to return the invariant distance, whereas in our approach we achieve this from the intrinsic properties of the algebra and a definition of a spacetime event in Eq. (10).

The representation of time with a Cartesian-type dimension in conventional approaches including STA, appears ill founded physically though due to the observed non-Cartesian like behavior of time, such as the time axis possessing a negative signature and the observed inability to freely move within the time dimension as is possible with space dimensions. Recall that although time is usually described by a positive Cartesian axis, it has a negative contribution to the Pythagorean distance in this space. Our approach on the other hand requires a minimal two dimensional Euclidean space, without the need for an imposed mixed metric structure, as the invariant spacetime interval arises naturally from the properties of the algebra, with the four-vectors and tensors typically employed in special relativity replaced with the multivector, thus requiring only a single Lorentz transformation operator, which also allows Lorentz covariance to be more easily ascertained. Also, with time now modeled as a bivector we find an algebraic structure that more appropriately models the nature of time.

Clifford’s geometric algebra was first published in 1873, extending the work of Grassman and Hamilton, creating a single unified real mathematical framework over Cartesian space, which naturally included the algebraic properties of scalars, complex numbers, quaternions and vectors into a single entity, called the multivector [23]. We find that this general algebraic entity, as part of a real two-dimensional Clifford algebra 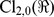, provides a natural alternative to a planar Minkowski vector space 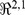 [24], [25].

### Two-dimensional Clifford Algebra

In order to describe a planar space, Clifford defined two algebraic elements 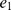 and 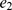, with the product rule.
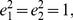
(1)with the composite element 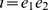, denoted by the Greek letter iota, being anticommuting, that is 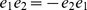, and assuming associativity squares to minus one [23], that is, 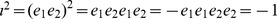, and hence can be used as an alternative to the scalar imaginary 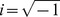 as a representation for the square root of minus one. A general Clifford multivector can be written through combining the various algebraic elements, as
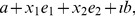
(2)where 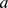 and 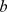 are real scalars, 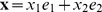 represents a planar vector, with 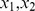 real scalars, and 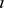 is the bivector, defining an associative non-commuting algebra. Denoting ∧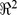 as the exterior algebra of 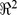 which produces the space of multivectors 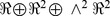, a four-dimensional real vector space denoted by 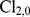.

#### Geometric product

A key property of Clifford’s algebra, is given by the product of two vectors, which are special cases of multivectors defined in Eq. (2). Given the vectors 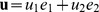 and 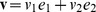, then using the distributive law for multiplication over addition, as assumed for an algebraic field, we find.
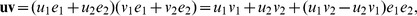
(3)using the properties defined in Eq. (1). We identify 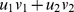 as the dot product and 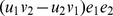 as the wedge product, giving



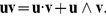
(4)Hence the algebraic product of two vectors produces a union of the dot and wedge products, with the significant advantage that this product now has an inverse operation. For 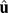 and 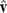 unit vectors, we have 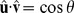 and 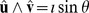, we therefore have 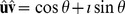, where 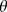 is the angle between the two vectors.

We can see from Eq. (4), that for the case of a vector multiplied by itself, that the wedge product will be zero and hence the square of a vector 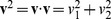, becomes a scalar quantity. Hence the Pythagorean length of a vector is simply 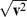, and so we can find the inverse vector.
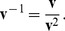
(5)


We define the distance measure or metric over the space as the scalar part of the geometric product, which for the special case of two vectors reduces to the dot product as shown in Eq. (3).

#### Rotations in space

Euler’s formula for complex numbers, carries over unchanged for the bivector 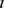, with which we define a rotor.
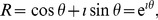
(6)which produces a rotation by 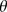 on the 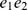 plane, in the same way as rotations on the Argand diagram. For example, for a unit vector 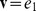 along the 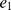 axis, acting with the rotor from the right we find 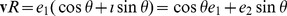, thus describing an anti-clockwise rotation by 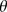. If we alternatively act from the left with the rotor, we will find a clockwise rotation by 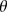.

However, we now show, that a rotation can be described more generally as a sequence of two reflections. Given a vector 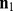 normal to a reflecting surface, with an incident ray given by 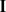, then we find the reflected ray [23].
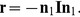
(7)


If we apply a second reflection, with a unit normal 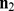, then we have.
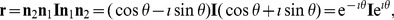
(8)using Eq. (4) for two unit vectors. If the two normals 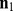 and 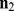 are parallel, then no rotation is produced. In fact the rotation produced is twice the angle between the two normals.

Hence rotations are naturally produced by conjugation, where if we seek to rotate a vector 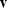 by an angle 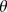, we calculate.
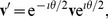
(9)which rotates in an anticlockwise direction. The rotation formula in Eq. (9) above, can in two-space, be simplified to a single right acting operator 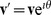. However this simplification is only possible in two-dimensions for the special case of rotations on vectors, and will be incorrect when applied to other algebraic elements or to vectors in higher dimensions, and hence Eq. (9) is the preferred way to apply operators such as rotors on vectors and multivectors.

## Results

### Clifford Multivectors as a Framework for Space and Time

Considering Minkowski’s definition of spacetime coordinates and Eq. (2), we describe planar spacetime events as the multivector.
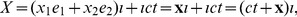
(10)with 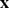 representing the position vector in the plane and 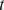 the observer time. This is without loss of generality for planar collisions, as we can always orientate this plane to lie in the plane of the relative velocity vector between the frames, and special relativity only requires two axes, the orthogonal and parallel directions to the relative velocity vector. The interpretation of a coordinate in Eq. (10) is the conventional one, of an observer moving through a preconfigured coordinate system, which at each point has a properly synchronized clock, from which the moving observer can read off the other frames local time 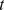 and position 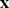 [16]. We then find the spacetime interval to be
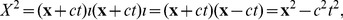
(11)using the fact that 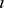 anticommutes with each component of 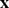, and 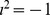, giving the correct spacetime distance. It is of interest to note that a modified spacetime coordinate given by 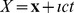 will also give the invariant spacetime distance as shown in Eq. (11), however using the definition in Eq. (10), we find that both the spacetime coordinates and the electromagnetic field have the identical Lorentz transformation, as well as enabling us to provide a unified description of the Dirac and Maxwell equations, shown later in Eq. (47) and Eq. (48).

We have from Eq. (10) the multivector differential.
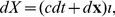
(12)which is independent of space and time translations as required by the principle of relativity and so can describe the larger Poincaré group. For the rest frame of the particle we have 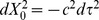, where we define in this case 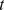 to represent the proper time 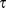 of the particle. We have assumed that the speed 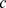 is the same in the rest and the moving frame, as required by Einstein’s second postulate. Now, if the spacetime interval defined in Eq. (11) is invariant under the Lorentz transformations defined later in Eq. (23), then we can equate the rest frame interval to the moving frame interval, giving
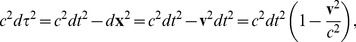
(13)with 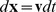, and hence, taking the square root, we find the time dilation formula 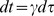 where



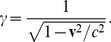
(14)From Eq. (12), we can now calculate the proper velocity, differentiating with respect to the proper time, giving the velocity multivector.
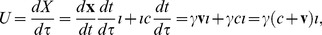
(15)where we use 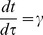 and 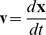. We then find



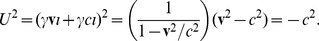
(16)We define the momentum multivector.
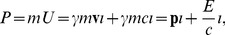
(17)with the relativistic momentum 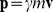 and the total energy 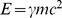.

Now, as 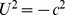, then 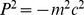 is an invariant between frames describing the conservation of momentum and energy, which gives.
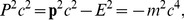
(18)the relativistic expression for the conservation of momentum-energy. The square of the velocity multivector resolving to a constant 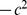 gives the expected property for the acceleration multivector 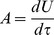, of being orthogonal the the velocity multivector, from
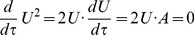
(19)using the chain rule from geometric calculus [26].

#### The lorentz group

The Lorentz transformations describe the transformations for observations between inertial systems in relative motion. The set of transformations describing rotations and boosts connected with the identity are described as proper and is referred to as the restricted Lorentz group described in four-dimensional spacetime as 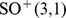, whereas if we also permit reflections we expand the transformations to the homogeneous Lorentz group 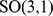. It is worth noting though that in two-dimensions reflections are also part of the restricted Lorentz group.

The most general transformation of a coordinate multivector is given by.
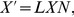
(20)where 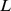 and 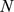 are general multivectors, with the coordinate multivector 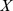 defined in Eq. (10). Requiring the invariance of the spacetime distance given by 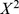 we find the relation
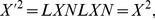
(21)which is satisfied if 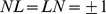. For a general multivector given by 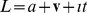, if we define the dagger operation 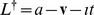, then we produce a scalar 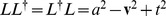. Hence in Eq. (21) we require 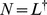 with 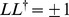. For the case 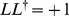, we can write 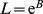, where 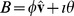, see Appendix S1, which describes a set of transformations connected with the identity. Though these transformations are not closed they nevertheless satisfy 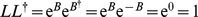 as required, using the fact that a multivector commutes with itself and naturally describes the Thomas rotation for two non-parallel boosts, that is 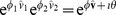. In order to close the operators consisting of general boosts and rotations we need to write 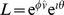. Other special transformations can be considered, such as with 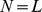 provided we enforce the condition 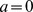, which then describes space and time reflections, so that we can write a unit multivector 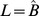, where 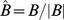 and 
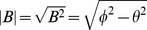
, giving 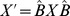, see Eq (7). The second general case 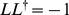 can be represented as 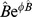 which is a combination of a proper Lorentz boost and a reflection and so not part of the restricted Lorentz group, but useful in representing collision processes with an associated energy transfer such as photons reflecting off electrons as in Compton scattering, described in Eq. (43).

The exponential of a multivector is defined by constructing the Taylor series.
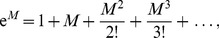
(22)which is absolutely convergent for all multivectors 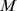 [18]. Also because of the closure of multivectors under addition and multiplication, we see that the exponential of a multivector, must also produce another multivector, and we find, in fact, a unique multivector 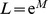, for each multivector 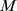 [18]. Hence in summary, all operators of the form
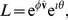
(23)applied to the multivector 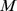 using the transformation
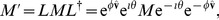
(24)will leave the spacetime distance invariant, defining the restricted Lorentz group [21]. We find for 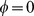 pure rotations as described by Eq. (9), and for 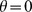, we find pure boosts, where 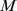 can denote the coordinate, momentum or electromagnetic field multivectors.

#### Spacetime boosts

Using the first component of the restricted Lorentz group defined in Eq. (23), operators of the form 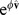, where the vector 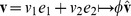, where 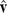 is a unit vector, with 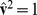, we find.
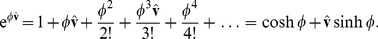
(25)


Transforming the spacetime coordinates 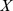 we find.
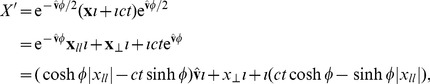
(26)where 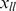 and 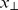 are the coordinates parallel and perpendicular respectively to the boost velocity direction 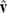, which is the conventional Lorentz boost, in terms of the rapidity 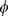, defined by 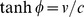, which can be rearranged to give 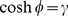 and 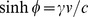. Substituting these relations we find
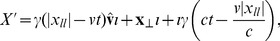
(27)which thus gives the transformation 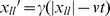, 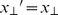 and 
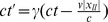
, the correct Lorentz boost of coordinates. The formula in Eq. (24) can be simply inverted to give 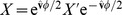, using the fact that 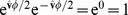. The relativity of simultaneity is a fundamental result of special relativity, and from the perspective of the Clifford multivector, Eq. (10), we see that it stems from the fact that during a boost operation, the terms for space 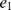 and 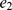 become mixed, resulting in the bivector term 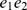, thus creating a variation in the observers time coordinate. Similarly the momentum multivector, shown in Eq. (17), will follow the same transformation law between frames shown in Eq. (24), with 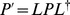. Serendipitously, we also find that the Lorentz boost of electromagnetic fields is subject to the same operator as coordinate transformations given by Eq. (24).

Given a general electromagnetic field represented by the multivector 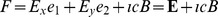, where for two-dimensional space we only have available a single magnetic field direction 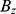 out of the plane, represented by the axial vector 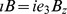. Applying the boost according to Eq. (24), with the exponentiation of a general boost vector 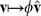, we find.
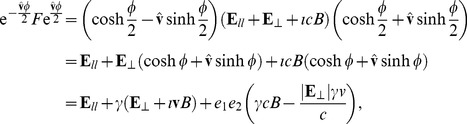
(28)which are the correct Lorentz transformations for an electromagnetic field. That is, the parallel field 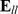 is unaffected, the perpendicular field 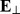 has been increased to 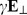 and the term 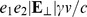, represents the 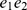 plane, also describable with an orthogonal vector 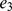 in three-space, hence this term gives the expected induced magnetic field 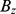 from the perpendicular electric field 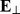.

Hence the exponential map of a Clifford multivector, naturally produces the restricted Lorentz transformations of spacetime coordinates and the electromagnetic field in the plane using the Lorentz boost Eq. (24), with the spacetime coordinate multivector given by Eq. (10) and the field multivector 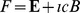.

#### Velocity addition rule

If we apply two consecutive parallel boosts, 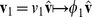 and 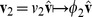, where 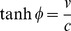, we have the combined boost operation.
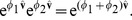
(29)


Hence we have a combined boost velocity.
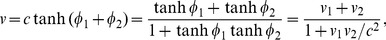
(30)the standard relativistic velocity addition formula. By inspection, the velocity addition formula implies that a velocity can never be boosted past the speed 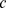, which confirms 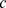 as a speed limit.

Hence, we have now demonstrated from the ansatz of the spacetime coordinate described by the multivector shown in Eq. (10), that we produce the correct Lorentz transformations, where the variable 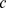 is indeed found to be an invariant speed limit. Numerically therefore, 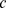 can be identified as the speed of light, since this is the only known physical object which travels at a fixed speed and represents a universal speed limit.

### Applications

#### 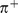-meson decay

A classic example of experimental confirmation for the special theory of relativity is its application to the decay of 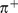-mesons, which are observed to enter the atmosphere at high velocity 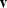 from outer space, having a known decay time at rest of 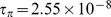 s, giving a spacetime coordinate multivector at rest of 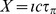. Boosting these coordinates to the 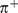-meson velocity, we have a boost 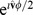, where 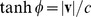, so we therefore find from Eq. (24).
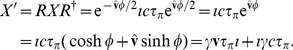
(31)


So that we have a decay time in laboratory coordinates of 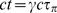, with a track length in the laboratory of 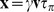, in agreement with experimental determinations [27].

#### Doppler shift

The Doppler shift of light, refers to the change of frequency caused by the relative velocity between source and observer. In the rest frame of the source, we can describe a single wavelength 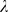 of emitted light using Eq. (10), setting up the 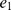 axis along the line of sight, as.
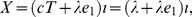
(32)where 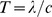 is the period of the wave, which gives 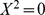 as required for a photon. We can describe an observer in relative motion with a boost in the 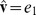 direction using 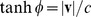, and we find from Eq. (24)



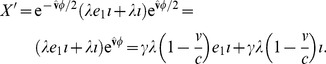
(33)So using the space (or alternatively time) component we find 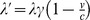 and using 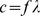 we find the standard relativistic Doppler shift formula.
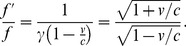
(34)


#### Thomas rotation

A surprising result occurs when we apply two non-parallel boosts, followed by their inverse boosts, in that the velocity of the frame does not return to zero. Furthermore, there is a rotation of the frame, called the Thomas rotation, a result, in fact, not noticed until 1925 [16].

For the case of two consecutive general boosts given by.
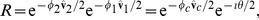
(35)where we use the results of Appendix S1, to write this in terms of a single combined boost 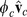 and a rotation 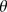, finding,
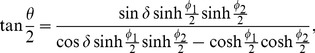
(36)where 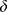 is the angle between the boost directions, given by 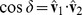. Hence we can see that only for parallel boosts, that is 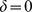, will there not in fact be a Thomas rotation, 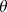, of the frame.

We can also write the Thomas rotation as a single exponential of a multivector.
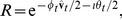
(37)using the results of Appendix S1.

#### Scattering processes

It is well established that energy and momentum conservation applies in relativistic dynamics, provided that the rest energy 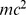 is now included along with the appropriate relativistic corrections, that is, defining momentum as 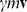, and the energy as 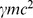. We now show that the two conservation laws can be bundled into a single momentum multivector defined in Eq. (17), giving a new perspective on momentum and energy conservation as the conservation of a multivector.

For example, if we are given a set of particles that are involved in an interaction, which then produce another set of particles as output. Then, in order to describe this collision interaction process we firstly include a separate momentum multivector for each particle, and then energy and momentum conservation between the initial and final states is defined by.
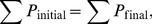
(38)assuming we are dealing with an isolated system. We know 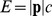 for a massless particle, so using Eq. (17) we write the momentum multivector for a photon as 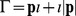, which gives 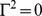 and for a massive particle 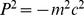 as shown in Eq. (18).

For Compton scattering, which involves an input photon striking an electron at rest, with the deflected photon and moving electron as products, we can write energy and momentum conservation using the multivectors as 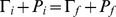, which we can rearrange to.
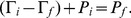
(39)


Squaring both sides we find.
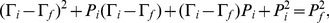
(40)remembering that in general the multivectors do not commute. Now, we have the generic results that 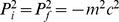 and 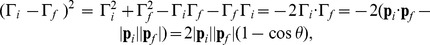 using 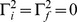. For the following two terms in Eq. (40), using 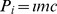, we have 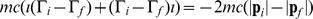. We therefore find from Eq. (40) that



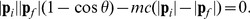
(41)Dividing through by 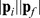 and substituting 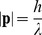 we find Compton’s well known formula.
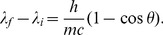
(42)


**Figure 1:**
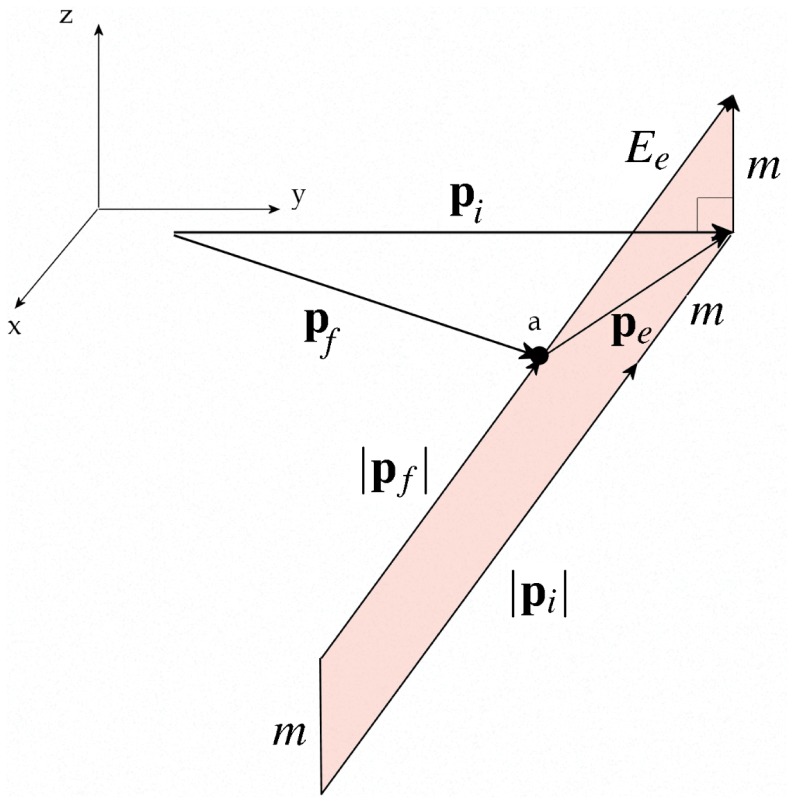
Graphical solution to Compton scattering (natural units with 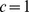). In order to obtain possible experimental outcomes the point 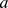 is moved in the plane of 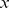 and 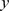, as shown, which automatically satisfies conservation of momentum given by the vector triangle, 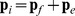 and the locus of points which also maintains the shape of the figure in the vertical plane as a parallelogram (shown in red) satisfies the conservation of energy. We have the Pythagorean distance giving the final energy of the electron 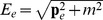, so that the requirement of a parallelogram implies the conservation of energy 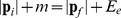. Hence this 3D graphical solution simultaneously satisfies the relativistic conservation of momentum and energy providing the solutions for Compton scattering.

The advantage of the momentum multivector is that energy and momentum conservation can be considered in unison as shown in Eq. (39), which also provides a clear solution path, whereas typical textbook methods rely on manipulating two separate equations describing momentum and energy conservation [27]. The multivector equation shown in Eq. (39) also leads to a graphical solution, shown in Fig. 1. This 3D visual model allows us to find a graphical solution simultaneously conserving relativistic momentum and energy.

We can also describe this process using GA as firstly the reflection of the photon off the electron, given by 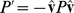 using Eq. (7), followed by a deboost of the photon due to the energy lost to the electron, given by the operator 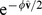, so that the new photon momentum multivector will be given by.
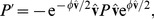
(43)where 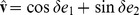 is the unit vector defining the direction of the electrons recoil with 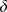 measured from the same axis as 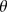, and 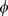 represents the amount of deboost of the photon, given by 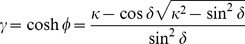, where 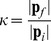 using 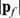 calculated from Eq. (42) using the relation 
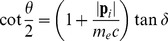
. While 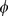 needs to be calculated using the analysis leading to Eq. (42), Eq. (43) nevertheless gives us an intuitive and coordinate free way to describe the photon in the Compton effect, as a reflection and deboost.

### Modeling Fundamental Particles with Multivectors

In the previous section we found that the momentum multivector provides a natural description for Compton scattering involving the interaction of photons and electrons, and so guided by the mathematical structure of the multivector we produce a simple model for the electron producing results consistent with special relativity. Using the multivector defined in Eq. (17), we can represent a particle moving with a velocity 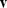 as.
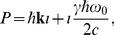
(44)where 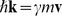. For a particle at rest, we therefore have 
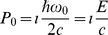
, where we use the de Broglie relation between total energy and frequency 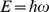, to find 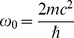. The bivector 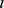 can be interpreted as a rotation operator, and so for a simplified semi-classical-type model, we can assume a circular periodic motion with a radius
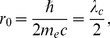
(45)where 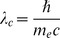 is the reduced Compton wavelength, which then gives the tangential velocity 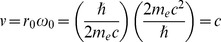 indicating an orbiting lightlike particle. This model leads to a natural explanation for time dilation, using the proper time invariant distance 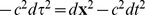, which can be rearranged to 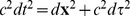, then because the proper time distance given by the circumference always moves perpendicular to the momentum vector, due to the bivector 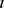 being perpendicular to the plane, then the net path distance of the lightlike particle, representing the observed time 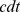 is simply the Pythagorean distance 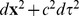 and because all photons are measured with the same speed according to special relativity, the period of the orbit will be increased by 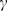 giving the expected time dilation effect.

We have now arrived at a model similar to previous elementary models of the electron developed by various authors [28]–[30]. The models are based on the *zitterbewegung* phenomena, first described by [31], an effect recently verified by experiment [32]–[34]. Schrödinger interpreted the *zitterbewegung* as arising from the interference of positive and negative energy states, but later described by [35] as a lightlike particle oscillating at the speed of light, with an amplitude equal to the reduced Compton wavelength.

In the footsteps of previous investigations [28]–[31], [35]–[38], a future development is to extend this work to three dimensional space.

### Wave Mechanics

A further application of the momentum multivector defined in Eq. (17), is through the standard substitutions 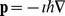 and 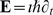, from which we produce the spacetime gradient operator as.
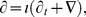
(46)where 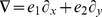 is the two-space gradient operator. We then find 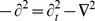 the d’Alembertian in two dimensions, so that 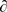 is a square root of the d’Alembertian. We therefore write for the Dirac equation
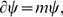
(47)where 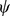 is a general multivector, shown in Eq. (2), which gives a Lorentz covariant equation isomorphic to the conventional Dirac equation in two dimensions (see Appendix S2), and comparable to the Dirac equation previously developed in three dimensional Clifford algebra [39], [40]. We can write Eq. (47) as 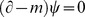, and acting from the left with 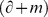, we produce 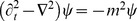, demonstrating that a solution of Eq. (47) is a solution of the Klein-Gordon equation. Adding an interaction with an electromagnetic potential we produce 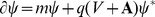, where 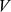 and 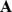 are the electromagnetic potentials and 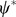 is a multivector with the sign flipped on the vector components.

Taking Eq. (47) with 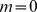 and adding a source multivector 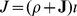, we can write.
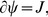
(48)which is isomorphic to Maxwell’s equations in two dimensions, provided we write the electromagnetic field as the multivector 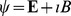 [40]. The square of the field produces the Lorentz invariant 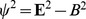. If we seek to complete the current multivector 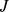 to a full multivector with a bivector term 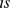, that is 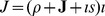, then we find that 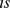 represents magnetic monopole sources. It is straightforward to show Lorentz covariance. Beginning with the primed frame we have from Eq. (48) 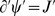. However we have 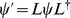 and 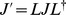, which implies
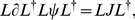
(49)which implies therefore implies 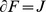, thus demonstrating covariance, using the property of the Lorentz transformation that 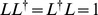.

If we calculate.
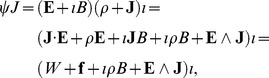
(50)then, inside the bracket, we find the work done by the field on the current 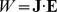 as a scalar and the vector force on the charges as 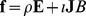, equivalent to 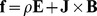 in three dimensions. We can write this in terms of the field alone through substituting Eq. (48), which gives
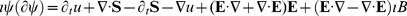
(51)where we have written 
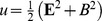
 representing the field energy and 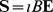 the Poynting vector in two dimensions. Inspecting expressions Eq. (50) and Eq. (51) we can see that it expresses the conservation of energy and momentum. In fact it is convenient to define a field momentum multivector
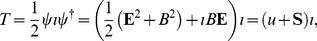
(52)which is in the form of a momentum multivector, as defined in Eq. (17). Now, we see that the first four terms in Eq. (51) can be expressed as 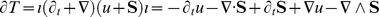, therefore we can express the conservation of energy as 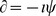 which gives 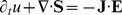, or Poynting’s theorem for the conservation of energy. The conservation of charge 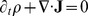 also follows from Maxwell’s equation through taking the divergence of Eq. (48).

A simple solution path is found through defining the field 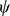 in terms of a multivector potential 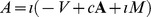, with 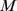 describing a possible monopole potential, given by 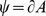. We then find Maxwell’s equations defined in Eq. (48) in terms of a potential becomes 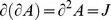 and because 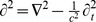 is a scalar differential operator we have succeeded in separating Maxwell’s equations into four independent inhomogeneous wave equations, given by the scalar, vector and bivector components of the multivectors, each with known solution.

For the Dirac equation, using the definition of Eq. (52) to define the Dirac current, we find defining a general Dirac wave function as 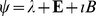, then.
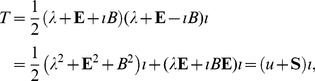
(53)then we find a positive definite density 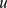 and a vector 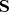. Then we find the divergence gives a conserved current 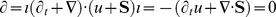 as required, now appearing as the conservation of energy. The Dirac equation for the plane has recently found application on the movement of electrons through graphene layers [41].

It is known that Einstein’s equations for general relativity describing gravity, if placed within a (2+1) spacetime, does not allow the propagation of gravitational waves as they require two orthogonal degrees of freedom orthogonal to the direction of propagation. Although, it should be noted, that Witten showed that the equations of GR can still describe the global topology of a (2+1) spacetime.

## Discussion

It is well established that Clifford’s geometric algebra, is a natural formalism suited for the study of geometrical operations of the plane, such as reflections and rotations [23]. However, we demonstrate additionally that spacetime represented as the Clifford multivector, as shown in Eq. (10), is a natural alternative to Minkowski spacetime, producing the correct spacetime interval and the required Lorentz transformation, directly from the properties of the algebra. Also the use of the momentum multivector defined in Eq. (17) allows the principle of momentum and energy conservation to be interpreted as the conservation of a multivector. We also find that the momentum multivector leads to a unified description of the Dirac and Maxwell’s equations in the plane. The mathematical structure of the wave multivector in Eq. (17), also leads to a simple model for the internal structure of the electron in Eq. (44), in accordance with previous developments [28]–[30], [35]–[38].

**Figure 2:**
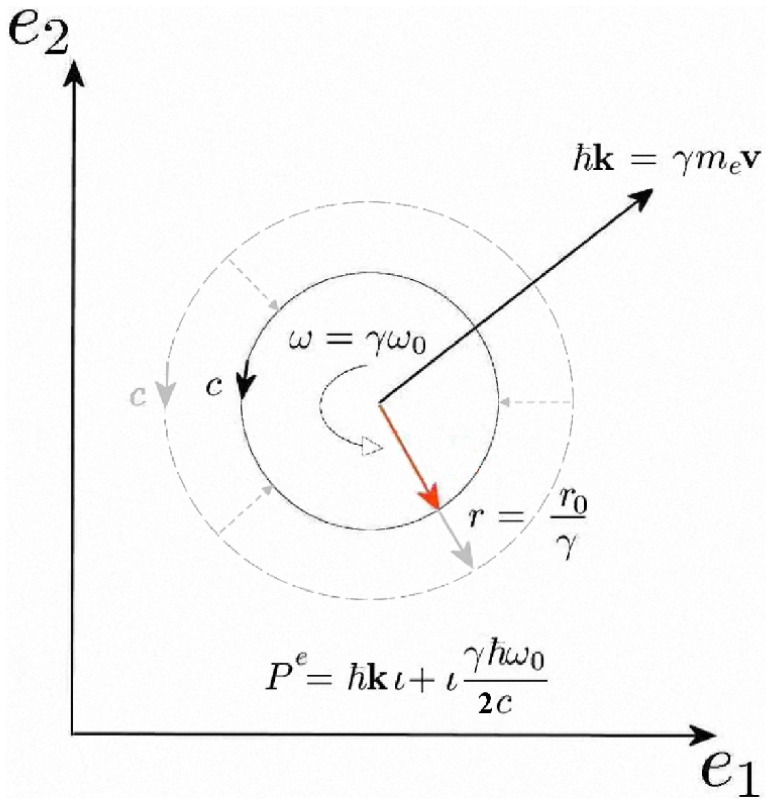
Multivector model for the electron, consisting of a light-like particle orbiting at the de Broglie angular frequency 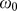
 **at a radius of** 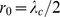 **in the rest frame, and when in motion described generally by the multivector** 
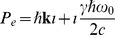
 **.** Under a boost, the de Broglie angular frequency will increase to 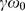, giving an apparent mass increase and time dilation, the electron radius will also shrink by 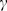, implying length contraction, thus naturally producing the key results of special relativity.

The definition of a spacetime event as a multivector in Eq. (10), also provides a new perspective on the nature of time, in that rather than being defined as an extra Euclidean-type dimension, it becomes instead a composite quantity of space, the bivector 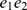. Minkowski’s famous quote is therefore particularly apt, *Henceforth space by itself, and time by itself, are doomed to fade away into mere shadows, and only a kind of union of the two will preserve an independent reality* [42]. As we have seen in Eq. (6), a bivector represents a rotation operator, and so it is natural to interpret time as an angular rotation at the de Broglie frequency 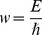 related to the frequency of the *zitterbewegung* [28], [31], [35], and encapsulated by a two dimensional model implied from the multivector description in Eq. (44), shown in Fig. 2.

A view of time as a rotational entity, has also been supported by recent experiments, which have identified a fluctuating electric field at the de Broglie frequency for an electron [43], and the use of the rotating electric field in circularly polarized light as an attosecond clock to probe atomic processes [44]–[47]. Hence the popular notion of time, as the ‘river of time’, certainly based in part on the pronouncement of Newton in the Principia, Book 1 [48], that time … *flows equably without relation to anything external …*, combined with time being promoted by Minkowski as a fourth dimension, may perhaps need to be amended to a description of a rotational entity, and adopting a water analogy, time would therefore be viewed descriptively as a whirlpool or an eddy current. Newton’s concept of the steady *flow of time* would relate in the multivector model to the constant spin rate at the de Broglie frequency of each particle that is constant in the particles’ rest frame, thus indeed *flowing equably*. Unforeseen by Newton though was the observed variation in this rotation rate with an *external* observer in relative motion, which produces the relativistic effects identified by Einstein.

The bivector describing time can also represent a unit area 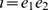, and Kepler’s second law of ‘equal areas in equal times’ can be written.
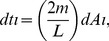
(54)where 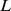 is the angular momentum, and 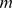 the mass of the satellite. So we can therefore reinterpret Kepler’s second law as a definition of time, forming a steady ‘tick’, independent of orbit ellipticity. Kepler’s law is in fact a restatement of the conservation of angular momentum for central force laws, through 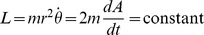 [49]. It is also now interesting to consider the impact on the nature of time if we expand the two-dimensional multivector in Eq. (10) to three dimensions. This firstly allows space vectors to possess three degrees of freedom 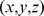, but also the single bivector 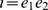 representing time will now expand to include the three bivectors of a three-dimensional multivector. Hence a direct implication of representing time as a bivector in two dimensions is that when expanding the model to three dimensions, time will now become three dimensional [50]–[53], associated with the three rotational degrees of freedom of three-dimensional space.

Minkowski spacetime diagrams, consisting of a space axis and a time axis are still applicable, though the time axis no longer represents a Euclidean time dimension, but simply shows the algebraic relationship between time as a bivector and space as a vector. The abstract nature of Minkowski diagrams are indeed confirmed by the rotation of the coordinate axes for a moving observer, which are tilted with respect to the original frame when displayed on the Minkowski diagram, a practice that is purely formal and not indicating a real rotation of the space or time axes between the frames [27]. Boosts are conventionally interpreted as rotations in time in comparison to rotations in space. However this interpretation needs to be revised from the new perspective of Clifford multivectors, with spatial rotations seen as bivector operators of the form 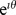 and boosts as vector operators of the form 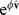.

There are many definitions of clock time possible, such as the rotation of the earth on its axis, or the vibration of a quartz crystal, however the one discussed here, based on the bivector rotation of particles is perhaps the most fundamental. The arrow of time is another property of time, however it has been recognized previously that this arises from the universe being far from equilibrium in a low entropy thermodynamic state. The steady progress towards high entropy as required by the second law of thermodynamics leading to the ‘heat death’ of the universe gives a perceived direction to time, though this is essentially unrelated to the definition of time given by the bivector rotation. The definition of time, as a bivector representing rotation, also allows the difficult concept of time beginning with the big bang to be more accessible as it now simply implies the non-existence of rotational degrees of freedom before the big bang. The creation of time with the big bang is in agreement with many philosophical conceptions of time, such as Augustine’s statement, *The world was made, not in time, but simultaneously with time* [54].

In summary, this approach from an abstract mathematical perspective based on the ansatz of spacetime represented by a Clifford multivector shown in Eq. (10), produces the correct spacetime metric and Lorentz transformations directly from the properties of the algebra, and thus similar to Minkowski’s approach, we explain the two postulates of Einstein based on the geometrical structure of spacetime. This systematic approach, is also shown to be advantageous in describing the Lorentz transformations, in that an exploration of the exponential map of a multivector, naturally produced rotations, boosts and the Thomas rotation of frames, and in fact the restricted Lorentz group represented simply as the multivector exponentials 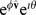. This Lorentz transform operator is generic, as it simultaneously provides the transformation for the coordinate, momentum-energy and electromagnetic fields, with all these objects modeled uniformly as multivectors. This can be compared with the conventional approach that uses four-vectors to represent coordinates and the momentum-energy but with a different structure, the antisymmetric field tensor, used to represent the electromagnetic fields, with necessarily different transformation operations for each type of object. Hence we see significant benefits with the use of multivectors as a description of spacetime, which allow the Lorentz transformations as well as the Dirac equation and Maxwell’s equations, to arise naturally in a simplified algebraic setting, without any unnecessary mathematical ‘overheads’, such as matrices, four-vectors, complex numbers, tensors or metric structures. It is hoped with the simplified two dimensional framework using only real numbers and two algebraic entities 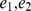, that a greater fundamental understanding of quantum mechanical processes at a fundamental level may be possible. The minimalist system that we have presented having just sufficient complexity to describe special relativity is therefore in line with Einstein’s ideal that: *It can scarcely be denied that the supreme goal of all theory is to make the irreducible basic elements as simple and as few as possible without having to surrender the adequate representation of a single datum of experience* [55].

## Supporting Information

Appendix S1 **Geometric Algebra.** Boost-rotation form of a multivector, the exponential of a general multivector and useful results from geometric calculus.
(PDF)

Appendix S2 **Wave Mechanics.** The Dirac equation in two dimensions, wave mechanics and Maxwell’s equations.
(PDF)
