# Supplementary material for: Revisiting Special Relativity: A Natural Algebraic Alternative to Minkowski Spacetime
Source: PLoS One. 2012 Dec 31;7(12):e51756. doi: 10.1371/journal.pone.0051756 (PMC3534154; doi:10.1371/journal.pone.0051756)
Supplement: Appendix S1 — Geometric Algebra. Boost-rotation form of a multivector, the exponential of a general multivector and useful results from geometric calculus. (PDF) [file pone.0051756.s001.pdf]

Supplementary Material

James M. Chappell, Azhar Iqbal, Nicolangelo Iannella, Derek Abbott

Results in Geometric Algebra

Boost-rotation form of a multivector

Given a general two-space multivector written as

$$M = r\cos\alpha + s\cos\beta e_1 + s\sin\beta e_2 + \iota r\sin\alpha, \tag{1}$$

then requiring $MM^\dagger = 1$, we find the condition $r^2 - s^2 = 1$. If we seek to write Eq. (1) in the exponential form

$$\begin{aligned}\rho \mathrm{e}^{\phi\hat{\mathbf{v}}}\mathrm{e}^{\theta\iota} &= \rho\Big(\cosh\phi\cos\theta + \sinh\phi(v_1\cos\theta - v_2\sin\theta)e_1 \\ &\quad + \sinh\phi(v_2\cos\theta + v_1\sin\theta)e_2 + \iota\cosh\phi\sin\theta\Big),\end{aligned} \tag{2}$$

consisting of a separate boost and rotation, we require

$$\begin{aligned}\rho &= \pm\sqrt{r^2 - s^2} = \pm 1,\ \theta = \alpha,\ \phi = \operatorname{arctanh}\left(\frac{s}{r}\right) = \operatorname{arctanh}\left(\frac{s}{\sqrt{1+s^2}}\right) \\ v_1 &= \cos(\beta - \alpha)\,,\ v_2 = \sin(\beta - \alpha),\end{aligned} \tag{3}$$

which are all well defined.

Exponential of a multivector

It is found that exponentiating the even subalgebra $a + \iota b$, which is a closed subalgebra, produces rotations and dilations, while exponentiating the vector $\mathbf{v} = v_1 e_1 + v_2 e_2$, produces Lorentz boosts. However the odd subalgebra is not closed and consequently the set of boosts is not closed but can also involve the Thomas rotation. However if we use the exponential of a full multivector, we encompass the set of non-parallel boosts and Thomas rotations.

Firstly, defining $B = \mathbf{v} + \iota b$, then $B^2 = (\mathbf{v} + \iota b)^2 = \mathbf{v}^2 - b^2 + b\mathbf{v}\iota + b\iota\mathbf{v} = \mathbf{v}^2 - b^2$, a scalar. We also have the result that $\mathrm{e}^{a+B} = \mathrm{e}^a\mathrm{e}^B$, as a is a scalar and hence commutes with B. Hence

$$\begin{aligned}\mathrm{e}^{a+\mathbf{v}+\iota b} &= \mathrm{e}^a\mathrm{e}^{\mathbf{v}+\iota b} \\ &= \mathrm{e}^a\left(1 + B + \frac{\mathbf{v}^2 - b^2}{2!} + \frac{B(\mathbf{v}^2 - b^2)}{3!} + \frac{(\mathbf{v}^2 - b^2)^2}{4!} + \dots\right) \\ &= \mathrm{e}^a\Bigg(1 + \frac{\mathbf{v}^2 - b^2}{2!} + \frac{(\mathbf{v}^2 - b^2)^2}{4!} + \dots \\ &\quad + \frac{B}{\sqrt{\mathbf{v}^2 - b^2}}\left(\sqrt{\mathbf{v}^2 - b^2} + \frac{\sqrt{\mathbf{v}^2 - b^2}(\mathbf{v}^2 - b^2)}{3!} + \dots\right)\Bigg) \\ &= \mathrm{e}^a\left(\cosh|B| + \hat{B}\sinh|B|\right),\end{aligned} \tag{4}$$

where $|B| = |\sqrt{\mathbf{v}^2 - b^2}|$, assuming $\mathbf{v}^2 > b^2$, and $\hat{B} = \frac{B}{|B|} = \frac{\mathbf{v}+\iota b}{|B|}$. If $v^2 < b^2$ we simply replace the hyperbolic trigonometric functions with trigonometric functions, and if $\mathbf{v}^2 = b^2$, then referring to the second line of the above derivation, we see that all terms following B are zero, and so, in this case $\mathrm{e}^{a+\mathbf{v}+\iota b} = \mathrm{e}^a(1 + \mathbf{v} + \iota b)$. Hence the exponential of a general multivector $\mathrm{e}^M = \mathrm{e}^{a+\mathbf{v}+\iota b}$ is well defined for all a, $\mathbf{v}$ and b. The reverse process, of finding the exponent for a given multivector, the logarithm of a multivector, is not always defined.

We are now in a position to classify the various transformation operators as shown in Table 1.

Operation L	Description	Comments
$e^{\iota\theta/2}$	Exp. of bivector, rotn by θ	Group of rotations
$e^{\phi\hat{\mathbf{v}}/2}e^{\iota\theta/2}$	Compound boost rotn.	Lorentz group connected with identity
$a+\mathbf{v}+\iota\theta$	General multivector	General boost, rotation, reflection, dilation
$e^{\phi\hat{\mathbf{v}}/2}$	Boost with $\tanh\phi = v/c$	Pure boosts
$e^{\phi\hat{\mathbf{v}}/2+\iota\theta/2}$	Exp. of multivector	Multiple boosts (Thomas rotn.)

Table 1. Classification of the Lorentz group, with the Lorentz transformation defined by $X' = LXL^\dagger$, with $LL^\dagger = \pm 1$. The first section forms a chain of subgroups, with each succeeding group having the previous groups as subgroups. The entries in the second section do not form a group, but are useful in describing pure boosts and compound boost situations.

Geometric calculus

The product rule for differentiation using Clifford variables

$$\nabla(AB) = e_i\partial_i(AB) = e_i(\partial_i A)B + e_i A(\partial_i B), \tag{5}$$

where we respect non-commutivity of the algebraic parts e_i of the differential operator.

The chain rule for a general function on a multivector can be expressed

$$\partial_x M(f) = \partial_f M \cdot \partial_x f, \tag{6}$$

where the dot product is specified on the RHS. For example for $M = \left(xe_1 + x^3 e_2\right)^2$ then defining $\mathbf{f} = xe_1 + x^3 e_2$, we have $M = \mathbf{f}^2$ and so we find

$$\partial_x M = 2\mathbf{f}\cdot\partial_x \mathbf{f} = 2(xe_1 + x^3 e_2)\cdot(e_1 + 3x^2 e_2) = 2x + 6x^5. \tag{7}$$

This can be checked by expanding $M(\mathbf{f}) = \mathbf{f}^2 = x^2 + x^6$ and then $\partial_x M = 2x + 6x^5$ as required.

We can generalize the chain rule for the gradient operator

$$\nabla M(f) = \dot{\nabla}\left(\partial_f M \cdot \dot{f}\right), \tag{8}$$

for example, for $M = \mathbf{E}^2$, we have

$$\nabla\left(\mathbf{E}^2\right) = \dot{\nabla}\left(2\mathbf{E}\cdot\dot{\mathbf{E}}\right) = 2\left(\nabla\cdot\mathbf{E} - \mathbf{E}\wedge\nabla\right)\mathbf{E}. \tag{9}$$

This can be confirmed by expanding the following expressions

$$\nabla\left(\mathbf{E}^2\right) = e_1\left(2E_x\partial_x E_x + 2E_y\partial_x E_y\right) + e_2\left(2E_x\partial_y E_x + 2E_y\partial_y E_y\right) \tag{10}$$

$$2\left(\nabla\cdot\mathbf{E}\right)\mathbf{E} = e_1\left(2E_x\partial_x E_x + 2E_x\partial_y E_y\right) + e_2\left(2E_y\partial_x E_x + 2E_y\partial_y E_y\right) \tag{11}$$

$$2\left(\mathbf{E}\wedge\nabla\right)\mathbf{E} = e_1\left(2E_x\partial_y E_y - 2E_y\partial_x E_y\right) + e_2\left(2E_y\partial_x E_x - 2E_x\partial_y E_x\right). \tag{12}$$

We also have for the gradient operator

$$\nabla\cdot(f\mathbf{E}) = (\nabla f)\cdot\mathbf{E} + f\nabla\cdot\mathbf{E} \tag{13}$$

$$\nabla(f\mathbf{E}) = (\nabla f)\mathbf{E} + f\nabla\mathbf{E}. \tag{14}$$

The following expressions are also useful

$$\mathbf{w}\cdot(\iota\mathbf{E}) = (\mathbf{w}\iota)\cdot\mathbf{E} \tag{15}$$

$$\mathbf{E}\wedge(\mathbf{w}\iota) = (\iota\mathbf{E})\wedge\mathbf{w} = \iota(\mathbf{E}\cdot\mathbf{w}). \tag{16}$$
