# Supplementary material for: Revisiting Special Relativity: A Natural Algebraic Alternative to Minkowski Spacetime
Source: PLoS One. 2012 Dec 31;7(12):e51756. doi: 10.1371/journal.pone.0051756 (PMC3534154; doi:10.1371/journal.pone.0051756)
Supplement: Appendix S2 — Wave Mechanics. The Dirac equation in two dimensions, wave mechanics and Maxwell’s equations. (PDF) [file pone.0051756.s002.pdf]

Supplementary Material

James M. Chappell, Azhar Iqbal, Nicolangelo Iannella, Derek Abbott

Wave Mechanics

The Dirac equation in two dimensions

We now aim to describe a form of the Dirac equation in Clifford algebra isomorphic to the conventional Dirac equation

$$\gamma^\mu \partial_\mu |\psi\rangle + iq\gamma^\mu A_\mu |\psi\rangle = -im|\psi\rangle, \tag{1}$$

where $i = \sqrt{-1}$ and γ^μ are the Dirac matrices, using natural units in which $c = \hbar = 1$. If we reduce the number of spatial dimensions to two, then the Dirac algebra can fit within the Pauli algebra, and we can write the Dirac equation as

$$\partial_t |\psi\rangle + (\sigma_1 \partial_x + \sigma_2 \partial_y) |\psi\rangle = -i\sigma_3 m |\psi\rangle, \tag{2}$$

where $\sigma_1, \sigma_2, \sigma_3$ are the Pauli matrices [1]. Naturally the one-dimensional Dirac equation can be found by ignoring the y direction as $\partial_t |\psi\rangle + \sigma_1 \partial_x |\psi\rangle = -i\sigma_3 m |\psi\rangle$.

If we select a spinor mapping to the two dimensional multivector as

$$|\psi\rangle = \begin{bmatrix} a_0 + ja_3 \\ a_2 + ja_1 \end{bmatrix} \leftrightarrow \psi = a_0 + a_2 e_1 + a_1 e_2 + a_3 e_1 e_2, \tag{3}$$

then we find the following mapping for the Pauli matrices

$$\sigma_k |\psi\rangle \leftrightarrow e_k \psi \tag{4}$$

for $k = 1, 2$ and

$$i\sigma_3 |\psi\rangle = \sigma_1 \sigma_2 |\psi\rangle \leftrightarrow e_1 e_2 \psi \tag{5}$$

using $iI = \sigma_1 \sigma_2 \sigma_3$. Expanding Eq. (2) we find

$$-\partial_t \psi = \partial_x \sigma_1 \psi + \partial_y \sigma_2 \psi + m \sigma_1 \sigma_2 \psi \tag{6}$$

using the relation $i\sigma_3 = \sigma_1 \sigma_2$. Mapping this to the multivector defined in Eq. (3) we find

$$-\partial_t \psi = e_1 \partial_x \psi + e_2 \partial_y \psi + m e_1 e_2 \psi. \tag{7}$$

Multiplying from the left by $-e_1 e_2$ we find

$$e_1 e_2 (\partial_t + \nabla) \psi = m\psi. \tag{8}$$

Hence defining $\partial = \iota (\partial_t + \nabla)$, where $\iota = e_1 e_2$, we find

$$\partial \psi = m\psi \tag{9}$$

where the Dirac wavefunction is described by the multivector in Eq. (3). We then find $-\partial^2 = \partial_t^2 - \nabla^2$ the d'Alembertian, thus allowing us to recover the Klein-Gordon equation in two dimensions from Eq. (9). This equation implies we have a Hamiltonian $H = -\iota\nabla + m = \mathbf{p} + m$. The one dimensional Dirac equation is also given by Eq. (9) but with the spatial gradient operator $\nabla = e_1 \partial_x$. Selecting a different permutation of Pauli matrices we can find an alternate equation $(\iota\partial_t - \nabla)\psi = m\psi$. The two versions of the Dirac equation corresponds to two possible square roots of the Klein-Gordon equation in two dimensions.

Bilinear observables

Given $\psi = \lambda + \mathbf{E} + \iota B$, then

$$\psi\tilde{\psi} = (\lambda + \mathbf{E} + \iota B)\,(\lambda + \mathbf{E} - \iota B) = \lambda^2 + \mathbf{E}^2 + B^2 + 2(\lambda\mathbf{E} + \iota B\mathbf{E}) = \rho + \mathbf{v}, \tag{10}$$

where $\tilde{\psi} = \lambda + \mathbf{E} - \iota B$ is the reversion operation. We therefore define the probability current as

$$J = \psi\tilde{\psi}\iota = \psi\iota\psi^\dagger = (\rho + \mathbf{v})\,\iota. \tag{11}$$

This is in the form of the four-velocity detailed in the main paper. The term $\rho = \lambda^2 + \mathbf{E}^2 + B^2$ is a positive definite scalar equivalent to $\rho = |\psi|^2 = \langle\psi|\psi\rangle$ conventionally calculated for the probability density. Thus this equation relates the Dirac current J to the wavefunction ψ. For $\psi \rightarrow \psi S$, we find

$$J = \psi S \iota S^\dagger \psi^\dagger \tag{12}$$

so that provided $S\iota S^\dagger = \iota$, S represents a gauge transformation. Multiplying from the right by S and remembering that $S^\dagger S$ is a scalar, then we find $S\iota = \iota S$ which implies that S commutes with ι and hence $S = \mathrm{e}^{\iota\theta} = \cos\theta + \iota\sin\theta$ describing a rotation in the spin plane $\iota = e_1 e_2$.

Calculating the divergence of our probability current

$$\partial \cdot J = \iota\,(\partial_t + \nabla)\cdot(\rho + \mathbf{v})\,\iota = -\partial_t\rho - \nabla\cdot\mathbf{v} = 0 \tag{13}$$

which is recognizable expression for conservation of charge or probability. Now with this definition of current we find

$$\begin{aligned} \nabla\cdot\mathbf{v} &= \nabla\cdot 2(\lambda\mathbf{E} + \iota B\mathbf{E}) \\ &= 2\,(\nabla\lambda)\cdot\mathbf{E} + 2\lambda\,(\nabla\cdot\mathbf{E}) + 2\,(\nabla B)\cdot(\iota\mathbf{E}) + 2B\,(\nabla\cdot\iota\mathbf{E}) \\ &= 2\,(\nabla\lambda)\cdot\mathbf{E} + 2\lambda\,(\nabla\cdot\mathbf{E}) - 2\mathbf{E}\cdot(\iota\nabla B) - 2\iota B\,(\nabla\wedge\mathbf{E})\,, \end{aligned} \tag{14}$$

where we have used $\mathbf{v}\cdot(\mathbf{w}\iota) = (\mathbf{v}\wedge\mathbf{w})\,\iota$ and $\mathbf{v}\cdot(\mathbf{w}\iota) = (\iota\mathbf{v})\cdot\mathbf{w}$.

To confirm our definition of probability current, we firstly write the Dirac equation and its reverse

$$\begin{aligned} \partial_t\psi &= -\nabla\psi - m\iota\psi \\ \partial_t\tilde{\psi} &= -\tilde{\psi}\nabla + m\tilde{\psi}\iota. \end{aligned} \tag{15}$$

Multiplying the first equation on the left with $\tilde{\psi}$ and the second equation on the right with ψ we obtain

$$\begin{aligned} \tilde{\psi}\,(\partial_t\psi) &= -\tilde{\psi}\,(\nabla\psi) - m\tilde{\psi}\iota\psi \\ \left(\partial_t\tilde{\psi}\right)\psi &= -\left(\tilde{\psi}\nabla\right)\psi + m\tilde{\psi}\iota\psi. \end{aligned} \tag{16}$$

Adding these two equations we find

$$\partial_t\left(\tilde{\psi}\psi\right) + \tilde{\psi}\,(\nabla\psi) + \left(\tilde{\psi}\nabla\right)\psi = 0. \tag{17}$$

Investigating the second and third terms, we find

$$\tilde{\psi}\,(\nabla\psi) = (\lambda + \mathbf{E} - \iota B)\nabla(\lambda + \mathbf{E} + \iota B) = (\lambda + \mathbf{E} - \iota B)\,(\nabla\lambda + \nabla\cdot\mathbf{E} + \nabla\wedge\mathbf{E} - \iota\nabla B) \tag{18}$$

$$\left(\tilde{\psi}\nabla\right)\psi = ((\lambda + \mathbf{E} - \iota B)\nabla)\,(\lambda + \mathbf{E} + \iota B) = (\nabla\lambda + \nabla\cdot\mathbf{E} - \nabla\wedge\mathbf{E} - \iota\nabla B)\,(\lambda + \mathbf{E} + \iota B) \tag{19}$$

The bivector terms $\lambda\nabla\wedge\mathbf{E}+\mathbf{E}\wedge\nabla\lambda-\mathbf{E}\wedge(\iota\nabla B)-\iota B\nabla\cdot\mathbf{E}$ cancel, leaving the scalar and vector components. We have the scalar parts

$$2(\nabla\lambda)\cdot\mathbf{E}+2\lambda(\nabla\cdot\mathbf{E})-2\mathbf{E}\cdot(\iota\nabla B)-2\iota B(\nabla\wedge\mathbf{E}) \tag{20}$$

which confirms our definition of probability current in Eq. (11) and Eq. (13), through comparison with Eq. (14).

We have the vector terms

$$\begin{aligned} & \partial_t\mathbf{v}+2\lambda\nabla\lambda+2\mathbf{E}(\nabla\cdot\mathbf{E})-2B\nabla B-2\lambda\iota\nabla B-2\iota B\nabla\lambda \\ = \ & \partial_t\mathbf{v}+\nabla\rho+2(\mathbf{E}\wedge\nabla)\mathbf{E}-2\nabla B^2-2\iota\nabla(\lambda B). \end{aligned} \tag{21}$$

Alternatively, using the Dirac equation and its reverse and multiplying the first equation on the right with $\tilde{\psi}$ and the second equation on the left with ψ we obtain

$$\begin{aligned} (\partial_t\psi)\tilde{\psi} &= -(\nabla\psi)\tilde{\psi}-m\iota\psi\tilde{\psi} \\ \psi\left(\partial_t\tilde{\psi}\right) &= -\psi\left(\tilde{\psi}\nabla\right)+m\psi\tilde{\psi}\iota. \end{aligned} \tag{22}$$

Adding these two equations we find

$$\partial_t\left(\psi\tilde{\psi}\right)+(\nabla\psi)\tilde{\psi}+\psi\left(\tilde{\psi}\nabla\right)+2\iota m\mathbf{v}=0. \tag{23}$$

Investigating the second and third terms, we find

$$(\nabla\psi)\tilde{\psi}=\nabla(\lambda+\mathbf{E}+\iota B)(\lambda+\mathbf{E}-\iota B)=(\nabla\lambda+\nabla\cdot\mathbf{E}+\nabla\wedge\mathbf{E}-\iota\nabla B)(\lambda+\mathbf{E}-\iota B) \tag{24}$$

$$\psi\left(\tilde{\psi}\nabla\right)=(\lambda+\mathbf{E}+\iota B)((\lambda+\mathbf{E}-\iota B)\nabla)=(\lambda+\mathbf{E}+\iota B)(\nabla\lambda+\nabla\cdot\mathbf{E}-\nabla\wedge\mathbf{E}-\iota\nabla B). \tag{25}$$

The bivector terms $\lambda\nabla\wedge\mathbf{E}+\mathbf{E}\wedge\nabla\lambda-\mathbf{E}\wedge(\iota\nabla B)-\iota B\nabla\cdot\mathbf{E}$ cancel, leaving the scalar and vector components. We have the scalar parts

$$2(\nabla\lambda)\cdot\mathbf{E}+2\lambda(\nabla\cdot\mathbf{E})-2\mathbf{E}\cdot(\iota\nabla B)-2\iota B(\nabla\wedge\mathbf{E}) \tag{26}$$

which confirms our definition of probability current in Eq. (11) and Eq. (13), through comparison with Eq. (14).

We have the vector terms

$$\begin{aligned} & \partial_t\mathbf{v}+2\lambda\nabla\lambda+2\mathbf{E}(\nabla\cdot\mathbf{E})+2B\nabla B+2\lambda\iota\nabla B-2\iota B\nabla\lambda+2\iota m\mathbf{v} \\ = \ & \partial_t\mathbf{v}+\nabla\rho+2(\mathbf{E}\wedge\nabla)\mathbf{E}+2\lambda\iota\nabla B-2\iota B\nabla\lambda+2\iota m(2(\lambda\mathbf{E}+\iota B\mathbf{E})) \end{aligned} \tag{27}$$

using the result that $\frac{1}{2}\nabla\mathbf{E}^2=(\nabla\cdot\mathbf{E})\mathbf{E}-(\mathbf{E}\wedge\nabla)\mathbf{E}$.

We have the Poynting vector $\mathbf{s}=\iota B\mathbf{E}$ which is equivalent so $S=\mathbf{E}\times\mathbf{B}$ in three dimensions. We have the energy density $u=\frac{1}{2}\left(\mathbf{E}^2+B^2\right)$, so that

$$\begin{aligned} \iota\psi(\partial\psi) &= \iota(\mathbf{E}+\iota B)\iota(\partial_t+\nabla)(\mathbf{E}+\iota B) \\ &= (\mathbf{E}-\iota B)(\partial_t+\nabla)(\mathbf{E}+\iota B) \\ &= \mathbf{E}\partial_t\mathbf{E}+B\partial_t B-\iota B(\nabla\wedge\mathbf{E})+(\iota\mathbf{E})\cdot\nabla B-\iota\partial_t B\mathbf{E}-\iota B\partial_t\mathbf{E}-B\nabla B+(\nabla\cdot\mathbf{E})\mathbf{E} \\ &\quad +\mathbf{E}\nabla\wedge\mathbf{E}-\iota B\nabla\cdot\mathbf{E}-\mathbf{E}\wedge(\iota\nabla B) \\ &= \frac{1}{2}\left(\partial_t\mathbf{E}^2+\partial_t B^2\right)+B\nabla\cdot(\iota\mathbf{E})+\nabla B\cdot(\iota\mathbf{E})-\partial_t(\iota B\mathbf{E})-\frac{1}{2}\left(\nabla B^2+\nabla\mathbf{E}^2\right) \\ &\quad -(\mathbf{E}\wedge\nabla)\mathbf{E}+2(\nabla\cdot\mathbf{E})\mathbf{E}-(\nabla\wedge\mathbf{E})\mathbf{E}-\iota(\nabla\cdot\mathbf{E})B+\iota(\mathbf{E}\cdot\nabla)B \\ &= \partial_t u+\nabla\cdot\mathbf{S}-\partial_t\mathbf{S}-\nabla u+(\mathbf{E}\cdot\nabla+\nabla\cdot\mathbf{E})\mathbf{E}+(\mathbf{E}\cdot\nabla-\nabla\cdot\mathbf{E})\iota B. \end{aligned} \tag{28}$$

Plane wave solution

We take a trial solution

$$\psi(X) = C\mathrm{e}^{\iota K\cdot X} = C\mathrm{e}^{\iota(\mathbf{k}\cdot\mathbf{x}-wt)}, \tag{29}$$

where C is some constant multivector, then on substitution into Eq. (61), we find

$$\begin{aligned}(\partial_t + c\boldsymbol{\nabla})\, C\mathrm{e}^{\iota(\mathbf{k}\cdot\mathbf{x}-wt)} &= C(-\iota w)\mathrm{e}^{\iota K\cdot X} + ce_1 C\iota k_x \mathrm{e}^{\iota K\cdot X} + ce_2 C\iota k_y \mathrm{e}^{\iota K\cdot X} \\ &= -wC\iota\mathrm{e}^{\iota K\cdot X} + c\mathbf{k}C\iota\mathrm{e}^{\iota K\cdot X} \\ &= -wC\iota\mathrm{e}^{\iota K\cdot X} + c\mathbf{k}C\iota\mathrm{e}^{\iota K\cdot X} \\ &= \frac{-\iota mc^2}{\hbar} C\mathrm{e}^{\iota K\cdot X}.\end{aligned} \tag{30}$$

Multiplying from the right by $\hbar\mathrm{e}^{-\iota K\cdot X}$, we find $(\hbar w - c\hbar\mathbf{k})\, C\iota = mc^2\, \iota C$. We thus need to satisfy

$$(\hbar w - c\hbar\mathbf{k})\, C + mc^2\iota C\iota = 0. \tag{31}$$

Given the multivector $C = a + \mathbf{u} + \iota b$, we find $\iota C\iota = -a + \mathbf{u} - \iota b$. Hence we have the equation

$$(\hbar w - c\hbar\mathbf{k})\,(a + \mathbf{u} + \iota b) = mc^2\,(a - \mathbf{u} + \iota b)\,. \tag{32}$$

For a particle at rest we have $E = \hbar w = mc^2$, giving

$$E\,(a + \mathbf{u} + \iota b) = mc^2\,(a - \mathbf{u} + \iota b)\,. \tag{33}$$

Hence for positive energy solutions we require $C = a + \iota b$, and for negative energy solutions with $E = -\hbar w$ we require $C = \mathbf{u}$. Hence we have the positive and negative wavefunctions $\psi^+ = (a+\iota b)\mathrm{e}^{-\iota wt}$ and $\psi^- = \mathbf{u}\mathrm{e}^{-\iota wt}$. The positive wavefunction acting on vector will rotate it clockwise and the negative wave function will rotate a vector in the negative direction in agreement with the de Broglie formula $E = \hbar w$. Hence this model gives immediately the result that a negative energy, is a negative angular frequency which implies a negative time direction, with an inverted spin, in agreement with Feynman's interpretation of negative energies as particles traveling back in time with an inverted spin.

For the general case we need to equate scalar, vector and bivector components of Eq. (32) to find

$$\begin{aligned}-c\mathbf{u}\cdot\mathbf{p} + (\hbar w - mc^2)a &= 0 \\ -ac\mathbf{p} + (\hbar w + mc^2)\mathbf{u} + bc\iota\mathbf{p} &= 0 \\ b(\hbar w - mc^2)\iota + c\mathbf{u}\wedge\mathbf{p} &= 0.\end{aligned} \tag{34}$$

From the first and third equations we find

$$a = \frac{c\mathbf{p}\cdot\mathbf{u}}{E - mc^2}, \quad b = \frac{c\mathbf{u}\wedge\mathbf{p}}{E - mc^2}\iota \tag{35}$$

and substituting into the second equation gives

$$-\frac{c^2\mathbf{p}\cdot\mathbf{u}}{E - mc^2}\mathbf{p} + (E + mc^2)\mathbf{u} - \frac{c^2\mathbf{u}\wedge\mathbf{p}}{E - mc^2}\mathbf{p} = 0 \tag{36}$$

multiplying through by $E - mc^2$ gives

$$-c^2\mathbf{p}\cdot\mathbf{u}\mathbf{p} + c^2\mathbf{p}^2\mathbf{u} - c^2\mathbf{u}\wedge\mathbf{p}\mathbf{p} = 0 \tag{37}$$

using the fact that $E^2 - m^2c^4 = c^2\mathbf{p}^2$, which can then be written

$$-c^2\mathbf{u}\mathbf{p}\mathbf{p} + c^2\mathbf{p}^2\mathbf{u} \tag{38}$$

which is identically zero because using associativity $(\mathbf{u}\mathbf{p})\mathbf{p} = \mathbf{u}(\mathbf{p}\mathbf{p}) = \mathbf{u}\mathbf{p}^2 = \mathbf{p}^2\mathbf{u}$. Hence Eq.(35) is sufficient to satisfy solve Eq. (34) and that allows both signs of energy $E = \pm\hbar w$, and so we can write

$$C = a + \mathbf{u} + \iota b = \frac{c\mathbf{p}\cdot\mathbf{u}}{\pm E - mc^2} + \mathbf{u} + \frac{c\mathbf{p}\wedge\mathbf{u}}{\pm E - mc^2} = \frac{c\mathbf{p}\mathbf{u}}{\pm E - mc^2} + \mathbf{u} = \left(\frac{c\mathbf{p}}{\pm E - mc^2} + 1\right)\mathbf{u}, \tag{39}$$

and substituting into Eq. (29) we have finally

$$\psi = \left(\frac{c\mathbf{p}}{\pm E - mc^2} + 1\right)\mathbf{u}\mathrm{e}^{\iota(\mathbf{k}\cdot\mathbf{x} - wt)}. \tag{40}$$

Interpreted as an operator we can identify a rotation, reflection and a boost.

If we assume minimal coupling of the form $\mathbf{p} = -\iota\hbar\nabla - q\mathbf{A}$ and $\mathbf{E} = \iota\hbar\partial_t - qV$ then we find

$$\partial\psi = \frac{mc}{\hbar}\psi - \frac{q}{\hbar c}(V + c\mathbf{A})\iota\psi\iota = \frac{mc}{\hbar}\psi - \frac{q}{\hbar c}A\psi\iota, \tag{41}$$

where $A = (V + c\mathbf{A})\iota$ is the potential multivector corresponding to a four-potential. This definition for the potential is compatible with Maxwell's equations, using

$$F = -\partial A = -\iota\left(\frac{1}{c}\partial_t + \nabla\right)(V + c\mathbf{A})\,\iota = -\nabla V - \frac{\partial\mathbf{A}}{\partial t} + c\nabla\wedge\mathbf{A} + \frac{\partial V}{c\partial t} + c\nabla\cdot\mathbf{A} = \mathbf{E} + \iota cB, \tag{42}$$

where $\mathbf{E} = -\nabla V - \frac{\partial\mathbf{A}}{\partial t}$ and $\iota cB = c\nabla\wedge\mathbf{A}$ and $\frac{\partial V}{c\partial t} + c\nabla\cdot\mathbf{A} = 0$ is the Lorenz gauge, which produces Maxwell's equations in terms of electromagnetic potentials as $\partial^2\mathbf{A} = -J$, which are a set of three uncoupled Poisson equations. We find $F^2 = \mathbf{E}^2 - B^2$ and $J\cdot A = qV - q\mathbf{v}\cdot\mathbf{A}$, and so we have the field Lagrangian $\mathcal{L} = \frac{1}{2}F^2 + J\cdot A = \frac{1}{2}\left(\mathbf{E}^2 - B^2\right) - \rho V + \mathbf{J}\cdot\mathbf{A}$, with Lagrange's equations $\partial\left(\frac{\partial\mathcal{L}}{\partial(\partial A)}\right) - \frac{\partial\mathcal{L}}{\partial A} = 0$.

We find $T = F\iota F = -\left(\mathbf{E}^2 + B^2 + 2\iota B\mathbf{E}\right)\iota = -\left(U + \mathbf{S}\right)\iota$, where $U = \mathbf{E}^2 + B^2$ is the total field energy and $\mathbf{S} = 2\iota B\mathbf{E}$ is the Poynting vector. We can then write Poynting's energy conservation theorem as $\partial\cdot T = J\cdot F$.

Stationary state solutions

We assume $\mathbf{A}$ and V are time-independent and we look for stationary states solutions of the form

$$\psi(X) = \psi(\mathbf{r})\mathrm{e}^{-\iota wt} = \psi(\mathbf{r})\mathrm{e}^{-\iota Et/\hbar}, \tag{43}$$

and substituting into the Dirac equation in Eq. (41) we find

$$\begin{aligned}\hbar c\partial\psi &= \iota\hbar\left(\partial_t + c\nabla\right)\psi(\mathbf{r})\mathrm{e}^{-\iota wt} \\ &= \iota\hbar\psi(\mathbf{r})(-\iota w)\mathrm{e}^{-\iota wt} + \iota\hbar c\nabla\psi(\mathbf{r})\mathrm{e}^{-\iota wt} \\ &= mc^2\psi(\mathbf{r})\mathrm{e}^{-\iota wt} - q(V + c\mathbf{A})\iota\psi(\mathbf{r})\mathrm{e}^{-\iota wt}\iota.\end{aligned} \tag{44}$$

Multiplying from the right with $\mathrm{e}^{\iota wt}$, we find

$$E\psi + \hbar c\nabla\psi\iota = -mc^2\iota\psi\iota + q(V - c\mathbf{A})\psi. \tag{45}$$

Writing $\psi = a + \mathbf{v} + \iota b = \phi_A + e_1\phi_2 = \phi_A + \phi_B$ which splits into the even and odd parts of the multivector, where $\phi_A = a + \iota b$ and $\phi_B = e_1(c + \iota d)$, then we find two coupled equations

$$\begin{aligned}E\phi_A + \hbar c\nabla\phi_B\iota &= (qV + mc^2)\phi_A - qc\mathbf{A}\phi_B \\ E\phi_B + \hbar c\nabla\phi_A\iota &= (qV - mc^2)\phi_B - qc\mathbf{A}\phi_A,\end{aligned} \tag{46}$$

which correspond to conventional solutions [2]. From the second equation we find

$$\phi_B = -\frac{qc\mathbf{A}\phi_A + \hbar c\nabla\phi_A\iota}{E - qV + mc^2} = \frac{(-qc\mathbf{A} + \hbar c\iota\nabla)\phi_A}{E - qV + mc^2}, \tag{47}$$

remembering that ι commutes with ϕ_A as it is the even subalgebra.

Non-relativisitic form of the Dirac equation

For positive energy and non-relativistic speeds we have $qV << mc^2$ and $p = \hbar k << mc^2$ and so we have approximately

$$\phi_B = \frac{(-q\mathbf{A} + \hbar\iota\nabla)\phi_A}{2mc}, \tag{48}$$

so that $\phi_B \approx \frac{\hbar\mathbf{k}}{mc}\phi_A \approx \frac{\mathbf{v}}{c}\phi_A$ and so for non-relativistic speeds $\phi_B << \phi_A$. Letting $E = E' + mc^2$, where $E' << mc^2$, and substituting Eq. (48) back into Eq. (46) we find

$$(E' - qV)\phi_A = c\,(q\mathbf{A} + \hbar\iota\nabla)\,\frac{(q\mathbf{A} - \hbar\iota\nabla)}{2mc}\phi_A, \tag{49}$$

which expands to

$$\begin{aligned} 2m(E' - qV)\phi_A &= q^2\mathbf{A}^2\phi_A - \hbar^2\nabla^2\phi_A - q\hbar\mathbf{A}\iota\nabla\phi_A + q\hbar\iota\nabla\left(\mathbf{A}\phi_A\right) \\ &= -\hbar^2\nabla^2\phi_A + q^2\mathbf{A}^2\phi_A + q\hbar\iota(\mathbf{A}\cdot\nabla)\phi_A + q\hbar\iota(\mathbf{A}\wedge\nabla)\phi_A \\ &\quad + q\hbar\iota(\nabla\cdot\mathbf{A})\phi_A + q\hbar\iota(\nabla\wedge\mathbf{A})\phi_A + q\hbar\iota\dot{\nabla}\mathbf{A}\dot{\phi}_A \\ &= \left(-\hbar^2\nabla^2\phi_A + q^2\mathbf{A}^2 + q\hbar\iota\mathbf{A}\cdot\nabla + q\hbar\iota\nabla\cdot\mathbf{A}\right)\phi_A \\ &\quad + q\hbar\iota\left(\mathbf{A}\wedge\nabla + \nabla\wedge\mathbf{A}\right)\phi_A + q\hbar\iota\dot{\nabla}\mathbf{A}\dot{\phi}_A, \end{aligned} \tag{50}$$

using associativity $A\left(\nabla\phi_A\right) = \left(A\nabla\right)\phi_A$ and $\nabla\left(\mathbf{A}\phi_A\right) = \nabla\mathbf{A}\phi_A + \dot{\nabla}\mathbf{A}\dot{\phi}_A$. However $\dot{\nabla}\mathbf{A}\dot{\phi}_A = (\dot{\nabla}\cdot\mathbf{A} + \dot{\nabla}\wedge\mathbf{A})\dot{\phi}_A = \mathbf{A}\cdot\nabla\phi_A - \mathbf{A}\wedge\nabla\phi_A$, which gives a cancellation to

$$\begin{aligned} 2m(E' - qV)\phi_A &= \left(-\hbar^2\nabla^2\phi_A + q^2\mathbf{A}^2 + q\hbar\iota\mathbf{A}\cdot\nabla + q\hbar\iota\nabla\cdot\mathbf{A}\right)\phi_A \\ &\quad + q\hbar\iota\left(\nabla\wedge\mathbf{A}\right)\phi_A + q\hbar\iota\left(\mathbf{A}\cdot\nabla\right)\phi_A. \end{aligned} \tag{51}$$

Now in two dimensions $\iota B_z = \nabla\wedge A$

$$\begin{aligned} 2m(E' - qV)\phi_A &= \left(-\hbar^2\nabla^2\phi_A + q^2\mathbf{A}^2 + q\hbar\iota\mathbf{A}\cdot\nabla + q\hbar\iota\nabla\cdot\mathbf{A}\right)\phi_A \\ &\quad - q\hbar B_z\phi_A + q\hbar\iota\left(\mathbf{A}\cdot\nabla\right)\phi_A. \end{aligned} \tag{52}$$

The term in brackets in three dimensions factorizes to $(i\hbar\nabla + q\mathbf{A})^2 = -\hbar^2\nabla^2 + q\mathbf{A}^2 + q\hbar i\nabla\cdot\mathbf{A} + q\hbar i\mathbf{A}\cdot\nabla$, however in two dimensions the pseudoscalar is non-commuting and so this factorization is not possible. We can select the Coulomb gauge, $\nabla\cdot\mathbf{A} = 0$ and for an electron $q = -e$ to give

$$E'\phi_A = \left(\frac{1}{2m}(-\hbar^2\nabla^2 + e^2\mathbf{A}^2 - 2e\hbar\iota\mathbf{A}\cdot\nabla + e\hbar B_z) - eV\right)\phi_A, \tag{53}$$

which is identical to the Pauli equation in two-dimensions, typically written as

$$E'\psi_A = \left(\frac{1}{2m}(-\hbar^2\nabla^2 + e^2\mathbf{A}^2 - e\hbar\iota\mathbf{A}\cdot\nabla + e\hbar\sigma\cdot\mathbf{B}) - eV\right)\psi_A, \tag{54}$$

except for the extra factor of two on the $\mathbf{A}\cdot\nabla$ term, where ψ_A is the conventional Pauli spinor and $\boldsymbol{\sigma}$ is the three-vector of Pauli matrices. This could be an artifact of a two-dimensional form. As is well known the coefficient $\frac{e\hbar}{2m}$ in front of B_z, gives a spin gyromagnetic ratio $g_s = 2$ in close agreement with experiment.

If we select $\mathbf{A} = 0$ then Eq. (53) reduces to

$$\left(-\frac{\hbar^2}{2m}\nabla^2 - eV\right)\psi_A = E'\psi_A, \tag{55}$$

the Schrödinger equation in two dimensions.

The representation of basic physical equations in a two-dimensional Clifford algebra $\mathrm{Cl}_{2,0}$, representing a uniform positive signature, gains significance from the isomorphism

$$\mathrm{Cl}_{(n+2,0)} \approx \mathrm{Cl}_{(0,n)} \otimes \mathrm{Cl}_{(2,0)} \tag{56}$$

so that higher dimensional Clifford algebra can be constructed from the two-dimensional case. This result also leads to the well known Bott periodicity of period eight for real Clifford algebras [3].

The multivector model for the electron

In the main paper we represented a particle as $P = \hbar \mathbf{k}\iota + \iota \frac{\gamma \hbar \omega_0}{2c}$, so that under a boost, the frequency ω_0 will increase to $\omega = \gamma\omega_0$, with the radius required to shrink to $r = r_0/\gamma$, so that the tangential velocity $v = rw = \left(\frac{r_0}{\gamma}\right)\gamma w_0 = r_0 w_0 = c$, remains at the speed of light. Hence this simplified two-dimensional model in Fig. 1, indicates that under a boost, the de Broglie frequency will increase to $\gamma\omega_0$ implying an energy and hence a mass increase γm_0, the frequency increase also implies time dilation, and the shrinking radius producing length contraction, thus producing known relativistic effects.

Now because the wave vector term $\mathbf{k}\iota$ represents a momentum perpendicular to the direction of motion, we can identify it with the angular momentum of the lightlike particle with $p = E/c = \gamma\hbar w_0/(2c) = \gamma mc$, remembering $\mathbf{r}\cdot\mathbf{p} = 0$, we have the spin angular momentum $L = \mathbf{rp} = \mathbf{r}\wedge\mathbf{p} = [\mathbf{r},\mathbf{p}] = \iota\left(\frac{\hbar}{2\gamma mc}\right)(\gamma mc) = \iota\frac{\hbar}{2}$ which is invariant, as expected for a spin-$\frac{1}{2}$ particle.

Integrating the momentum multivector with respect to the proper time τ, remembering that $dt = \gamma d\tau$, and dividing by the rest mass m we find

$$X = \mathbf{x}\iota + \iota\frac{\hbar\theta_0}{2mc} = \mathbf{x}\iota + \iota r_0\theta_0, \tag{57}$$

where $\omega_0 = \frac{d\theta_0}{dt}$. Inspecting the bivector component, we find

$$r_0 d\theta_0 = r_0 w_0 dt = \left(\frac{\hbar}{2mc}\right)\left(\frac{2mc^2}{\hbar}\right) dt = cdt, \tag{58}$$

and hence the time can be identified as the circumferential distance $r_0 d\theta_0$ at half the Compton radius $\frac{\hbar}{2mc}$.

Wave mechanics

We now work within the two-dimensional Clifford multivector to intuitively produce two-dimensional versions of Dirac's and Maxwell's equations. From the de Broglie hypothesis that all matter has an associated wave [4], given by the relations $\mathbf{p} = \hbar\mathbf{k}$ and $E = \hbar w$, we finding the wave multivector

$$K = \frac{P}{\hbar} = \mathbf{k}\iota + \frac{w}{c}\iota = \left(\frac{w}{c} + \mathbf{k}\right)\iota. \tag{59}$$

We now find the dot product of the wave and spacetime multivectors $K\cdot X = \mathbf{k}\cdot\mathbf{x} - wt$, giving the phase of a traveling wave. Hence for a plane monochromatic wave we can write $\psi = \mathrm{e}^{\iota K\cdot X} = \mathrm{e}^{\iota(\mathbf{k}\cdot\mathbf{x} - wt)}$, which leads to the standard substitutions, $\mathbf{p} = -\iota\hbar\nabla$ and $\mathbf{E} = \iota\hbar\partial_t$ and so we define from the momentum multivector

$$\partial = -\frac{\iota}{\hbar}P = \frac{-\iota}{\hbar}\left(\mathbf{p}\iota + \frac{E}{c}\iota\right) = \iota\left(\frac{1}{c}\partial_t + \nabla\right), \tag{60}$$

where $\nabla = e_1\partial_x + e_2\partial_y$. We therefore find $-\partial^2 = \frac{1}{c^2}\partial_t^2 - \nabla^2$ the d'Alembertian in two dimensions, so that ∂ is the square root of the d'Alembertian. Following Dirac, we therefore write

$$\partial\psi = \frac{mc}{\hbar}\psi, \tag{61}$$

which is isomorphic to the conventional Dirac equation, and comparable to the Dirac equation previously developed in three dimensional Clifford algebra [5, 6]. Acting from the left a second time with the differential operator ∂ we produce the Klein-Gordon equation, $\left(\frac{1}{c^2}\partial_t^2 - \nabla^2\right)\psi = -\frac{m^2c^2}{\hbar^2}\psi$.

For the non-relativistic case, summing the kinetic and potential energy, we find the total energy $E = T + V = \frac{p^2}{2m} + V$, and substituting the standard operators for $\mathbf{p}$ and E we find

$$\left(-\frac{\hbar^2}{2m}\nabla^2 + V\right)\psi = \iota\hbar\partial_t\psi, \tag{62}$$

which for a multivector $\psi = a + \iota b$ produces the Schrödinger equation in two dimensions, remembering that ι commutes with scalars and so acts equivalently to the scalar imaginary $\sqrt{-1}$ in this case. The wave function represents a rotation in the plane e_1e_2 and therefore the Schrödinger equation as represented in Eq. (62) describes an eigenstate of spin in the plane e_1e_2.

Maxwell's equations

For a massless particle we have the Klein-Gordon equation $\partial^2\psi = 0$, with a solution $\partial\psi = 0$ from the Dirac equation in Eq. (61), however we can also write

$$\partial\psi = J, \tag{63}$$

where J is a general multivector. Acting a second time with the spacetime gradient produces

$$\partial^2\psi = \partial J \tag{64}$$

and provided $\partial J = 0$, we satisfy the massless Klein-Gordon equation. Now $\partial J = \partial \cdot J + \partial \wedge J$, and for $J = (\rho + \mathbf{J})\iota$ representing source currents, where we now switch to natural units with $c = \hbar = 1$, we find firstly

$$\partial \cdot J = \partial_t\rho + \nabla \cdot \mathbf{J} = 0 \tag{65}$$

which is the requirement of charge conservation. Also $\partial \wedge \mathbf{J} = \partial_t\mathbf{J} + \nabla \wedge \mathbf{J} + \nabla\rho$ that is also zero for steady currents and curl free sources, and hence for this restricted case Eq. (63) is also a solution to the Klein-Gordon equation. Then writing the electromagnetic field as the multivector $\psi = \mathbf{E} + \iota B$, we have produced Maxwell's equations, that is, from Eq. (63) we find

$$\left(\partial_t + \nabla\right)\left(\mathbf{E} + \iota B\right) = \rho - \mathbf{J} \tag{66}$$

that when expanded into scalar, vector and bivector components, gives $\boldsymbol{\nabla} \cdot \mathbf{E} = \rho$, $\iota\boldsymbol{\nabla}B - \frac{\partial\mathbf{E}}{\partial t} = \mathbf{J}$, and $-\iota\boldsymbol{\nabla} \wedge \mathbf{E} + \frac{\partial B}{\partial t} = 0$ respectively, and noting that in three dimensions $-\iota\boldsymbol{\nabla} \wedge \mathbf{E} = \nabla \times \mathbf{E}$ and $\iota\nabla B = \nabla \times \mathbf{B}$, we see that we have produced Maxwell's equations for the plane. This equation also very naturally expands to produce Maxwell's equations in three dimensions as $\left(\partial_t + \nabla\right)\left(\mathbf{E} + i\mathbf{B}\right) = \rho - \mathbf{J}$, where the magnetic field now becomes a three-vector, with $i = e_1e_2e_3$ the trivector [6].

Hence using a general multivector $\psi = \lambda + \mathbf{E} + \iota B$ we have in natural units

$$\begin{aligned} \partial^2\psi &= m^2\psi, \quad \text{Klein} - \text{Gordon equation} \\ \partial\psi &= m\psi, \quad \text{Dirac equation} \\ \partial\psi &= J, \quad \text{Maxwell's equations } (m = 0). \end{aligned} \tag{67}$$

That is, with the assumption of the form of the spacetime gradient ∂ given in Eq. (60) and the spacetime multivectors, the two simplest first order differential equations we can write are Maxwell's equations and the Dirac equation. Also, setting $m = 0$ in the Dirac equation we find $\partial\psi = 0$, the Weyl equation for the plane. Hence the two dimensional Clifford multivector provides a natural 'sandbox', or simplified setting, within which to explore the laws of physics. As a generalization, Maxwell's source term can be expanded to a full multivector to give $J = (\rho + \mathbf{J} + \iota s)\iota$, and s describes magnetic monopole sources.

Solutions

An elegant solution path is found for the Maxwell and Dirac equation in Eq. (67) through defining the field ψ in terms of a multivector potential $A = \iota\,(-V + c\mathbf{A} + \iota M)$, with M describing a possible monopole potential, given by

$$\psi = \partial A. \tag{68}$$

We then find Maxwell's equations defined in Eq. (67) in terms of a potential becomes

$$\partial^2 A = J. \tag{69}$$

Now, as $\partial^2 = \nabla^2 - \frac{1}{c^2}\partial_t^2$ is a scalar differential operator we have succeeded in separating Maxwell's equations into four independent inhomogeneous wave equations, given by the scalar, vector and bivector components of the multivectors, each of which have the well known solution [7] given by

$$A = \frac{\mu_0}{4\pi}\int_{vol}\frac{J}{r}d\tau, \tag{70}$$

where $r = |\mathbf{r} - \mathbf{s}|$, the distance from the field point $\mathbf{r}$ to the charge at $\mathbf{s}$, and where we calculate values at the retarded time. The field can then be found from Eq. (68) by differentiation.

We find $\iota(\partial_t + \nabla)\,(u + \mathbf{S})\,\iota = -\partial_t u - \nabla\cdot\mathbf{S} + \partial_t\mathbf{S} + \nabla u - \nabla\wedge\mathbf{S}$, therefore we can express the conservation of momentum and energy as

$$\partial T = -\iota\psi J + (\mathbf{E}\cdot\nabla + \nabla\cdot\mathbf{E})\mathbf{E} + 2\iota\nabla B\cdot\mathbf{E}, \tag{71}$$

where the scalar components express the conservation of energy and the vector components the conservation of momentum. The cumbersome $(\mathbf{E}\cdot\nabla + \nabla\cdot\mathbf{E})\mathbf{E}$ term is typically absorbed into a stress energy tensor [7]. The conservation of charge $\partial_t\rho + \nabla\cdot\mathbf{J} = 0$ also follows from Maxwell's equation through taking the divergence of Maxwell's equation $\partial\psi = J$.

We can also define a Lagrangian $L = \frac{1}{2}\epsilon_0\psi^2 - J\cdot A$, which through the Euler-Lagrange equations produces Maxwell's equation.

References

1. Thaller B (1992) The Dirac Equation. New York, NY (United States); Springer-Verlag.
2. Bransden BH, Joachain CJ (2000) Quantum Mechanics. Prentice Hall.
3. Gallier J (2005) Clifford algebras, clifford groups, and a generalization of the quaternions: The pin and spin groups. manuscript) Department of Computer and Information Science, University of Pennsylvania http://www cis upenn edu/˜ cis610/clifford pdf .
4. de Broglie L (1923) C.R. Hebd. Séanc. Acad. Sci., Paris, 177, 506, 548, 630. Ph.D. thesis, Thesis de Doctoral.

5. Hestenes D (2003) Mysteries and insights of Dirac theory. In: Annales de la Fondation Louis de Broglie. Fondation Louis de Broglie, volume 28, p. 367.

6. Boudet R (2008) Relativistic Transitions in the Hydrogenic Atoms: Elementary Theory, volume 52. Springer Verlag.

7. Griffiths DJ (1999) Introduction to Electrodynamics. Prentice Hall.

Figure Legends

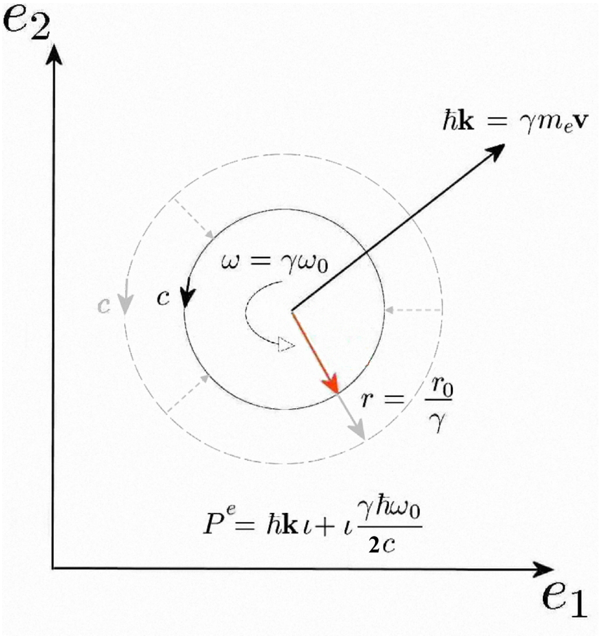

Figure 1. Multivector model for the electron, consisting of a light-like particle orbiting at the de Broglie angular frequency ω_0 at a radius of $r_0 = \lambda_c/2$ in the rest frame, and when in motion described generally by the multivector $P_e = \hbar\mathbf{k}\iota + \iota\frac{\gamma\hbar\omega_0}{2c}$. Under a boost, the de Broglie angular frequency will increase to $\gamma\omega_0$, giving an apparent mass increase and time dilation, the electron radius will also shrink by γ, implying length contraction, thus naturally producing the key results of special relativity.
